# On the Calibration of a Numerical Model for Concrete-to-Concrete Interface

**DOI:** 10.3390/ma14237204

**Published:** 2021-11-25

**Authors:** Sławomir Dudziak, Wioletta Jackiewicz-Rek, Zofia Kozyra

**Affiliations:** Institute of Building Engineering, Faculty of Civil Engineering, Warsaw University of Technology, Al. Armii Ludowej 16, 00-637 Warsaw, Poland; w.jackiewicz-rek@il.pw.edu.pl (W.J.-R.); z.kozyra@il.pw.edu.pl (Z.K.)

**Keywords:** concrete-to-concrete interface, cohesive elements, traction–separation law, Abaqus, fracture energy

## Abstract

The study was devoted to the numerical modelling of concrete-to-concrete interfaces. Such an interface can be found in many modern composite structures, so proper characterisation of its behaviour is of great importance. A strategy for calibration of a model based on cohesive finite elements and the elastic-damage traction–separation constitutive law available by default in the Abaqus code was proposed. Moreover, the default interface material model was enhanced with the user-field-variables subroutine to include a real strength envelope for such interfaces. Afterwards, the modelling approach was validated with numerical simulation of the most popular tests for determining the strength characteristics of concrete-to-concrete interfaces: three-point bending beam with a notch, splitting bi-material cubic specimens, and slant-shear tests. The results of own pilot studies were used as well as those reported by other researchers. The performed simulations proved the accuracy of the proposed modelling strategy (the mean ratio of ultimate forces obtained with numerical models and from experiments was equal to 1.01). Furthermore, the presented examples allowed us to better understand the basic test methods for concrete interfaces and the observed mechanisms of failure during them.

## 1. Introduction

Numerical modelling of concrete, cf. [1,2,3], and its contact zones with other buildings material is and will continue to be a current topic due to advances in concrete technology and material engineering [4]. In every reinforced concrete member, there are steel-to-concrete interfaces, which were thoroughly studied in reference [5]. The interfaces could also be found in composite structures as tubular columns filled with concrete [6] or in structures strengthened with composite sheets [7]. The last mentioned reference shows how effective a modern finite element (FE) system can be for structure assessment, since the failure modes and force-deflection curves for complex retrofitted beams were perfectly captured in this study.

**Figure 1:**
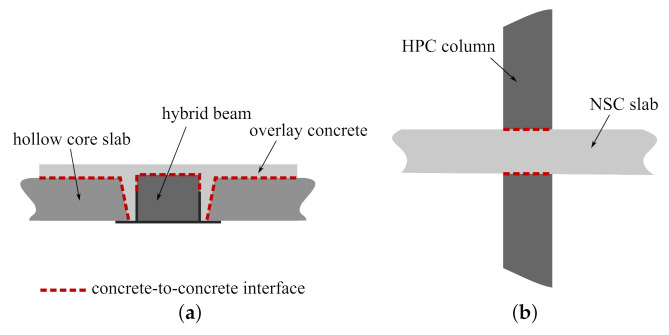
Examples of concrete-to-concrete interfaces in structures: (**a**) a composite floor on hybrid beams, (**b**) a slab-column connection.

The present study puts forward concrete-to concrete interfaces. Interfaces of different types of concretes, whose strength properties vary significantly, can often be found in modern structures, cf. Figure 1. For example, they occur in composite structures as slim-floors [8,9] or composite T-beams [10]. In the former, they influence the structure load capacity and deformability tremendously, since they are located at the interface between prefabricated hollow slabs, hybrid beams, and  cast in situ overlay concrete, so their total area is of great value. Such types of interfaces can also be found in high buildings at the connections between columns and floors [11]. The columns are usually made of high-performance concrete (HPC), whereas the concrete class of floor can be remarkably lower. A concrete-to-concrete interface is also formed as a result of structure strengthening by casting additional concrete layers [12].

Concrete-to-concrete interfaces usually have lower strength properties than the concrete layers that form it. Therefore, the proper and robust numerical model for the interface is crucial to simulate the behaviour of many composite structures. It has to precisely predict the strength of the interface (in a complex traction stress state, i.e., with normal and shear tractions) as well as its post-cracking behaviour. Such models are implemented in popular FE codes like ATENA [13], ANSYS [14], or Abaqus [15]. The calibration of an interface model in the first code, which is dedicated to the modelling of concrete structures, is easier than in the other two general-purpose FE systems. Especially, the standard damage initiation criterion in the ANSYS or Abaqus code is not suitable for concrete under a complex traction state.

**Figure 2:**
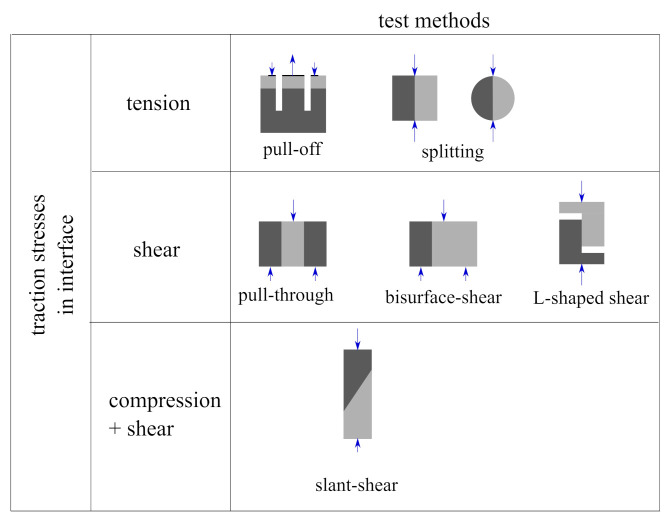
The most popular methods of testing concrete-to-concrete interfaces strength.

To determine the mechanical parameters of the interface model, the results of different strength tests can be used. They were widely described in many references [12,16,17,18,19]. In general, such tests aim to induce a homogenous traction stress state at the interface: tension or shear dominated (with compressive normal traction stresses as well). Schemes of the most popular test methods are shown in Figure 2. A pull-off test is often used in a quick assessment of bond strength [20] and can be used for in situ conditions as well. Modifications of standard laboratory tests for homogenous concrete like splitting or direct tension are also used for samples prepared in a laboratory [18,21]. In Figure 2, the most popular tests for determination of interface shear strength were presented [22,23], but new testing methods have also been developed [24]. The easiest way of testing the interface strength under compression and shear is a slant-shear test [25]. To determine the fracture energy of an interface, methods initially developed for homogenous concrete are used, like the three-point bending sample with a notch [26,27] or the wedge splitting test [28]. For a quick assessment of the strength properties of an interface, it is convenient to introduce the bond efficiency coefficient $\alpha_{int}$ [16,29] which can be calculated as follows:

(1) $$\alpha_{int}=\frac{f_{int}}{f_{w}},$$

where: $f_{int}$ and $f_{w}$ are the strength of the interface and the weaker concrete layer obtained from the same test method, respectively.

The bond strength between layers of concrete cast at different times is affected by many factors described in numerous references, cf. [16,25,30,31]. After the literature survey, the following factors can be considered as the most important: properties of the overlay and substrate concretes, surface characteristics (its cleanliness and mechanical preparation), compaction of concrete mix, and curing conditions, which influence shrinkage strains. The influence of the shrinkage is hard to capture with specimens of small dimensions, which was clearly presented in reference [21].

There are a few recent studies concerning the calibration of a numerical model for the concrete-to-concrete interface. Frenzel and Cubarch [32] performed shear tests of a concrete interface with regular, infra-lightweight, and foam concrete. On this basis, they calibrated a numerical model for a concrete-to-concrete interface subjected to pure shear in the ATENA code and obtained satisfactory agreement between the simulation and test results. Farzad et al. [33] conducted three types of tests: flexural, direct shear, and slant-shear ones, which enabled them to obtain strength characteristics of ordinary-to-ultra-high performance concrete (UHPC) interface in different traction stress states. Afterwards, they proposed parameters for the concrete damage plasticity model (CDP) in the Abaqus code for fictitious continuum material corresponding to the interface behaviour. However, such an approach is not recommended by the Abaqus manual [15]. Using continuum material models with an interface element is appropriate in the case of flexible interfaces (with lower stiffness than the stiffness of connected parts) and when it is possible to determine the strength and deformability of the material, which constitutes an interface in laboratory tests. Such a situation does not occur for a concrete-to-concrete interface—this kind of connection is obviously initially rigid, and it is impossible to extract its material due to its infinitesimal thickness. According to the manual [15], a material model based on a traction–separation relation should be used for such kinds of interfaces. Valikhani et al. [22] calibrated a model based on cohesive elements and the traction–separation law available in ATENA for a concrete-to-UHPC interface. Only shear loading was considered in this study. The latest study by Yuan et al. [34] concerns a rock-to-concrete interface. It contains detailed research on composite beams made of concrete and rock as well as the proposition of calibration of the traction–separation law for interface subjected to tension (mode I of fracture) implemented in the ANSYS software [14]. All in all, the authors of this study could not find in the literature a complete strategy for calibration of a concrete-to-concrete interface subjected to various loading conditions (combination of normal and tangential traction stresses), especially using cohesive elements and the traction–separation relation available by default in the Abaqus or ANSYS code.

The main aim of this study was to propose a complete strategy for calibration of a concrete-to-concrete interface according to the mentioned assumptions. Simulations were performed with the Abaqus code [15] using cohesive elements and the traction–separation-type material model. The standard constitutive model was enhanced with a simple USDFLD user subroutine coded in FORTRAN, which facilitates the correct prediction of the interface strength subjected to compressive normal forces. The issue of fracture energy in two modes of fracture (tensile and shear) was discussed and implemented in the analyses. The results of experimental tests found in the literature (i.e., in references [21,25,26]), as well as the results obtained during own pilot studies, were used to validate the proposed strategy. The validation part was divided into four case studies (CS). The following types of concrete-to-concrete interface laboratory tests were examined: three-point bending of a beam with a notch, splitting of cubic specimens, and slant-shear tests on prisms.

The major novel elements presented within the article are as follows: a complete strategy for calibration of interface traction–separation laws for concrete-to-concrete interfaces available by default in the Abaqus code and the detailed non-linear simulation of the most popular strength tests of concrete-to-concrete interfaces. They took into account: advanced constitutive models for concretes and their interfaces as well as detailed geometry of the loading devices. It is worth mentioning that the progress of delamination of bi-material specimens is hard to follow with modern optical measurement systems due to their brittle failure modes, so only numerical studies could give a wider insight into this process at this point [23,27].

## 2. Strength Test Used for Calibration

### 2.1. Notched Beam by Chen et al. (2021)

Chen et al. [26] performed a three-point bending test on high-strength and low-strength concrete and two types of their interface, namely, without special surface preparation and reinforced with steel fibres. The aim of the tests was to determine the fracture characteristics of two types of interfaces. Beams of dimensions: 150 × 150 × 550 mm with a notch of 50 mm high were investigated. For each concrete, the cubic compressive ($f_{cm,cube}$), flexural ($f_{ctm,flex}$), and splitting ($f_{ctm,split}$) tensile strength were determined as well as fracture parameters like the fracture energy ($G_{f}$) and the fracture toughness. The most important results of these laboratory tests, important for the present studies, are summarized in Table 1. The bond effectiveness coefficient $\alpha_{int}$ for the interface can be estimated at 0.46. The values of fracture energies seemed to be quite high—their values were greater than the ones predicted by code formulas [35,36]. The obtained values of the fracture energy for homogenous specimens were also greater than the one reported by Shah and Chandra Kishen [37]. They obtained $G_{f}=157÷172$ N/m for the concrete of similar strength as the low-strength concrete ($f_{cm}=34$ MPa) and using a very similar testing procedure. On the other hand, values $G_{f}$ for the interfaces were close to each other (≈70÷80 N/m in reference [37]).

### 2.2. Own Splitting and Shear-Slant Tests

A pilot study, which facilitates the determination strength of the interface, was performed at the Faculty of Civil Engineering WUT. The research program consisted of slant-shear and splitting tests. The slant-shear specimens were of a rectangular shape with dimensions 100 × 100 × 500 mm, and the shear plane was placed at 30 degrees with the vertical direction. The splitting tests were performed using cubic specimens of 150 mm edge. The assumed class for the stronger concrete was C30/37 (concrete A) and for the stronger one was C55/67 (concrete B). The mix composition was summarized in Table 2.

**Figure 3:**
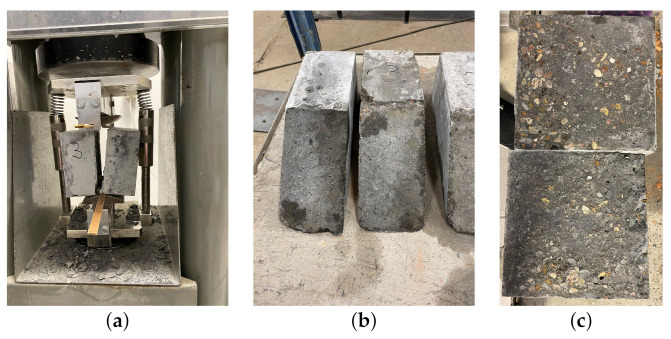
(**a**) The test stand after a splitting test. (**b**) Examples of surfaces after slant-shear tests. (**c**) Examples of surfaces after splitting tests.

During each casting, six additional 150 mm cubic specimens were prepared in order to determine the cubic compressive (three specimens) and splitting tensile strength (three specimens). The cubic compressive and tensile strengths were determined in accordance with their standards, respectively, [38,39]. Firstly, concrete B was cast (substrate), then, after 7 days, concrete A was poured into the mould (overlay). Before casting the weaker concrete, the contact surface of the hardened half of the specimen was subjected to chipping with a manual hammer. The specimens were cured in water up to the strength tests in order to reduce the influence of shrinkage. The strength tests were performed after 28 days since the second casting. The results of the tests are summarised in Table 3 and Table 4. The mean values, the standard deviation (STD), and the coefficient of variation (CoV) were reported. The specimens after splitting and shear-slant tests are shown in Figure 3. In case of slant-shear, the pure adhesive failure mode was obtained for all specimens, whereas the specimens in splitting the test presented a mixed failure mode (adhesive–cohesive). The bond efficiency coefficient was quite high (0.88) due to the early age of the substrate concrete and the surface preparation technique. The coefficient of variation for the tests of the interface was much higher than for homogenous specimens, which is consistent with other reported research data [16,25].

The following well-known formula for determining the splitting tensile strength was used:

(2) $$f_{ctm,split}=\frac{2F_{ult}}{\pi \;L\;d}\;,$$

where: $F_{ult}$—the ultimate force, L=150mm—the length of load line, and d=150mm—the length of sample destruction cross-section. The relations between the ultimate load and strength characteristics of an interface ($\sigma_{ult}$—the ultimate compressive normal traction, $\tau_{ultimate}$—the ultimate shear traction) are as follows [25]:

(3) $$\sigma_{ult}=\frac{F_{ult}\sin^{2}\left(\alpha \right)}{a^{2}}\;\tau_{ult}=\frac{F_{ult}\sin\left(\alpha \right)\cos\left(\alpha \right)}{a^{2}}\;,$$

where: $\alpha =30^{\circ }$—the interface angle with the vertical, and a=100mm—the length of the cross-section edge.

### 2.3. Splitting and Shear Slant Tests by Santos and Julio (2009, 2011)

Santos and Julio performed the wide laboratory test programme, which aimed at assessing the shear strength between concrete layers [21,25]. They analysed the influence of factors such as the surface preparation, shrinkage, the difference of ages, and concrete stiffness. For the purpose of the present studies, specimens from series L28 and substrate surface-treated with wire-brushing (WB) were chosen. In this series, the added concrete was cast after 28 days. The same two types of tests were performed like in the pilot studies—the splitting and slant-shear tests. To determine the compressive (three cubes) and splitting tensile (five cubes) strength, standard cubes of edge length equal to 150 mm and five specimens with dimensions 150 × 150 × 450 mm were used during slant-shear tests. The splitting test was modified by using a flat surface in order to load two parts of composite specimens more evenly. The most important outcomes of the laboratory tests are summarised in Table 5 and Table 6.

## 3. Numerical Modelling Strategy

**Figure 4:**
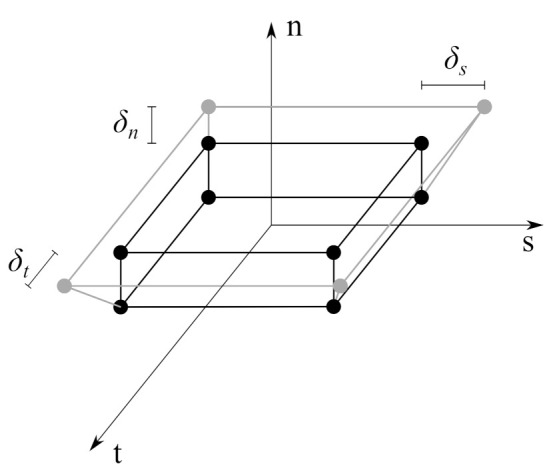
Cohesive element—local coordinate system and separations. The initial configuration is shown with black lines and the deformed one with grey lines.

The interface was modelled using cohesive elements, whose symbol is COH3D8 according to programme documentation [15]—see Figure 4. Such elements have eight nodes, so they use a linear approximation of the displacement field inside the element. They can be used with continuum-based constitutive relations or with a constitutive model formulated in terms of a traction–separation function.

In a 3D domain in the **elastic regime** (before damage initiation), the traction–separation law neglecting normal-shear states coupling has the following form:

(4) $$t=\left[\begin{array}{c} t_{n}\\ t_{s}\\ t_{t} \end{array}\right]=\left[\begin{array}{ccc} K_{nn} & 0 & 0\\ 0 & K_{ss} & 0\\ 0 & 0 & K_{tt} \end{array}\right]\;\left[\begin{array}{c} \epsilon_{n}\\ \epsilon_{s}\\ \epsilon_{t} \end{array}\right]=K\;\epsilon ,$$

where: t—the traction stress vector; indexes *n*, *s*, and *t* mean normal, first, and second tangential direction, respectively, e.g., $t_{n}$—traction normal to the interface; $K_{nn}$, $K_{ss}$, and $K_{tt}$—the stiffness of interface material in three directions, $\epsilon_{n}=\frac{\delta_{n}}{T_{0}}$, $\epsilon_{s}=\frac{\delta_{s}}{T_{0}}$, and $\epsilon_{t}=\frac{\delta_{t}}{T_{0}}$, respectively—interface nominal strains, $T_{0}$—initial thickness of the interface, $\delta_{n}$, $\delta_{s}$, and $\delta_{t}$—separations (displacement jumps at an interface) in three orthogonal directions. It is worth mentioning that the traction stress vector is related to the Cauchy stress tensor (σ) in the following manner t=σn, where n is normal to the interface plane [40,41].

Equation (4) can be rewritten as:

(5) $$t=\left[\begin{array}{c} t_{n}\\ t_{s}\\ t_{t} \end{array}\right]=\left[\begin{array}{ccc} K_{nn}^{\prime } & 0 & 0\\ 0 & K_{ss}^{\prime } & 0\\ 0 & 0 & K_{tt}^{\prime } \end{array}\right]\;\left[\begin{array}{c} \delta_{n}\\ \delta_{s}\\ \delta_{t} \end{array}\right],$$

where: $K_{nn}^{\prime }=\frac{K_{nn}}{T_{0}}$, $K_{ss}^{\prime }=\frac{K_{ss}}{T_{0}}$, and $K_{tt}^{\prime }=\frac{K_{tt}}{T_{0}}$—which are stiffness factors of the interface. In such a form, the elastic part of constitutive model for interface is given in references [13,40]. The stiffness coefficients for concrete–concrete interfaces have no clear physical interpretation since the interface before degradation can be assumed as a rigid one. Some authors try to prescribe a $T_{0}$ physical interpretation, e.g., the interface zone was assumed as a 100 μm thickness in reference [33]. However, assuming very small interface thickness, resulting in large values of its stiffness, can have an adverse influence on the convergence of the incremental-iterative procedure due to the large values of the unbalanced force vector. On the other hand, the introduction of too-small values can result in the wrong prediction of stress concentrations, which occur in the vicinity of two material connections. Therefore, the recommendations from the ATENA manual seem to be reasonable [13]. It recommend there to assume the following stiffness for the cohesive elements for the concrete–concrete interface:

(6) $$K_{nn}^{\prime }\approx \frac{E}{0.005\;a}\;K_{ss}^{\prime }=K_{tt}^{\prime }\approx \frac{G}{0.005\;a},$$

where: *a*—the biggest dimension of the connected parts and *E* and *G* are Young’s and Kirchhoff’s modules of the weaker concrete, respectively.

The inelastic part of the traction–separation law implemented in the Abaqus code was formulated in the framework of the damage theory. The onset to the damage state is indicated by the **damage initiation criterion**. Among the few available ones, the quadratic nominal stress criterion was selected. It can be represented as:

(7) $${\left(\frac{\langle t_{n}\rangle }{t_{n}^{max}}\right)}^{2}+{\left(\frac{t_{s}}{t_{s}^{max}}\right)}^{2}+{\left(\frac{t_{t}}{t_{t}^{max}}\right)}^{2}=1,$$

where: $t_{n}^{max}$—maximum normal traction stress (i.e., tensile strength, associated with mode I in the nomenclature of fracture mechanics); $t_{s}^{max}$ and $t_{t}^{max}$—maximal shear tractions in directions *n* and *s* (i.e., shear strength in pure shear), respectively. 〈…〉—the Macaulay brackets, which means the following operation:

(8) $$\langle x\rangle =\left\{\begin{array}{cc} x & \mathrm{if}\;x\geq 0\\ 0 & \mathrm{if}\;x<0 \end{array}\right..$$

**Figure 5:**
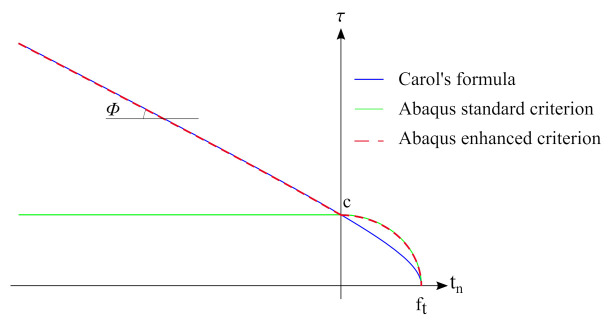
Failure envelopes for a concrete-to-concrete interface.

Therefore, damage factors are not activated in a pure compression state, and allowable shear stresses do not depend on normal stress—see Figure 5. Such behaviour does not correspond to the concrete–concrete interface, since compressive normal stress causes an increase in the shear strength. Carol’s formula [42] is believed to describe the failure envelope for the concrete—concrete interface correctly [18,40,43]:

(9) $$\tau^{ult}\left(t_{n}\right)=\sqrt{{\left(c-t_{n}\tan\left(\phi \right)\right)}^{2}-{\left(c-f_{t}\tan\left(\phi \right)\right)}^{2}},$$

where: $\tau =\sqrt{t_{s}^{2}+t_{t}^{2}}$—the resultant shear traction; $f_{t}$—the tensile strength of interface; and *c*—the cohesion. The standard Abaqus damage traction–separation interface model was enhanced by a user subroutine USDFLD to make the failure envelope pressure-dependent:

(10) $${\left(\frac{\langle t_{n}\rangle }{f_{ctm}}\right)}^{2}+{\left(\frac{t_{s}}{\tau^{\prime ult}\left(t_{n}\right)}\right)}^{2}+{\left(\frac{t_{t}}{\tau^{\prime ult}\left(t_{n}\right)}\right)}^{2}=1,$$

where: $\tau^{\prime ult}\left(t_{n}\right)=\left\{\begin{array}{cc} f_{sh} & \mathrm{if}\;t_{n}\geq \;0\\ \tau^{ult}\left(t_{n}\right) & \mathrm{if}\;t_{n}<0 \end{array}\right.$, $f_{ctm}$ and $f_{sh}$—the tensile and shear strength, respectively.

The USDFLD subroutine allowed us to modify the parameters of models implemented in the Abaqus code by default at the beginning of each increment of the Newton scheme. Consequently, it is important to set a small value of increment load size when one uses such an approach. On the other hand, the problem is highly non-linear, so small increments are necessary for the convergence of the incremental-iterative procedure. The proposed algorithm reads the normal traction and calculates the admissible shear stress according to Carol’s formula. All the discussed criteria are shown in Figure 5.

The last issue connected with a traction–separation interface model is its **post-cracking behaviour**. After reaching the damage-initiation criterion, tractions are calculated taking into account the damage factor *D*:

(11) $$\begin{array}{c} t_{n}=\left\{\begin{array}{ccc} \left(1-D\right)K_{nn}\;\epsilon_{n} & \mathrm{if} & \epsilon_{n}\geq 0\\ K_{nn}\;\epsilon_{n} & \mathrm{if} & \epsilon_{n}<0 \end{array}\right.\\ t_{s}=\left(1-D\right)K_{ss}\;\epsilon_{s}\\ t_{t}=\left(1-D\right)K_{ss}\;\epsilon_{t} \end{array}$$

The above equation shows that in the case of interface compression, there is no reduction in normal stiffness. The evolution of the damage parameter under a combination of normal and shear deformation is described using an effective displacement:

(12) $$\delta_{m}=\sqrt{{\langle \delta_{n}\rangle }^{2}+\delta_{s}^{2}+\delta_{t}^{2}}\;.$$

**Figure 6:**
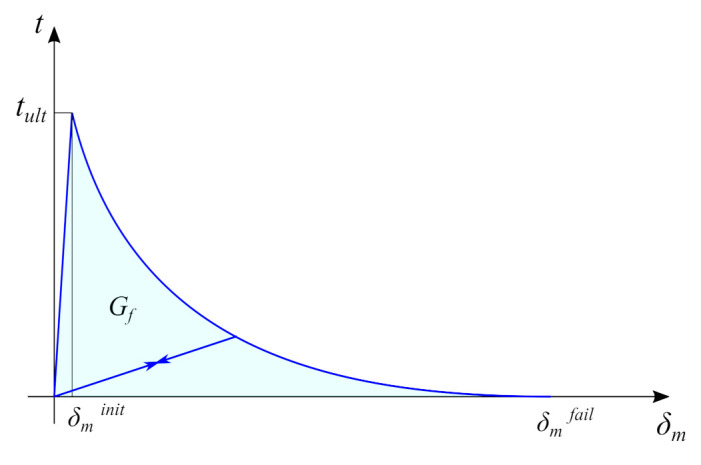
The plot of implemented traction–separation law. $t=\sqrt{{\langle t_{n}\rangle }^{2}+t_{s}^{2}+t_{t}^{2}}$.

In the present modelling strategy, exponential damage evolution was used, in which damage variable *D* is described by the relation:

(13) $$D\left(\delta_{m}^{max}\right)=1-{\left(\frac{\delta_{m}^{init}}{\delta_{m}^{max}}\right)}^{2}\left(1-\frac{1-\exp\left(-\alpha \frac{\delta_{m}^{max}-\delta_{m}^{init}}{\delta_{m}^{fail}-\delta_{m}^{init}}\right)}{1-\exp(-\alpha )}\right),$$

where: $\delta_{m}^{max}$—the maximum effective displacement during the loading history; $\delta_{m}^{init}$—the effective displacement at the damage initiation; $\delta_{m}^{fail}$—the effective displacement at complete failure; and α—the parameter which controls the rate of softening (in the present studies, the typical assumed value for concrete was equal to 7.0 [44]). The model behaviour is shown in Figure 6.

The post-peak behaviour of the concrete-to-concrete interface differed significantly for tension and shear states. In the present study, the effective displacement at failure was related to the fracture energy for the mode I type of fracture ($G_{f,I}$) or for mode II ($G_{f,II}$). The energy dissipated in any mode ($G_{f}$) can be calculated according to:

(14) $$G_{f}=\frac{1}{2}t^{ult}\delta_{m}^{init}+\int_{\delta_{m}^{init}}^{\delta_{m}^{fail}}\frac{t^{ult}}{\delta_{m}^{init}}\left(1-D\right)\;\delta \;d\delta ,$$

where: $t^{ult}$—the ultimate stress in the given mode. The first term can be neglected due to its very small value in comparison to the second one. After calculating the integral and some mathematical manipulations, the formula for the effective displacement of failure can be obtained:

(15) $$\delta_{m}^{fail}=\frac{G_{f}\;\alpha \left(\exp\left(\alpha \right)-1\right)}{t^{ult}\left(\exp\left(\alpha \right)-\alpha -1\right)}$$

**Figure 7:**
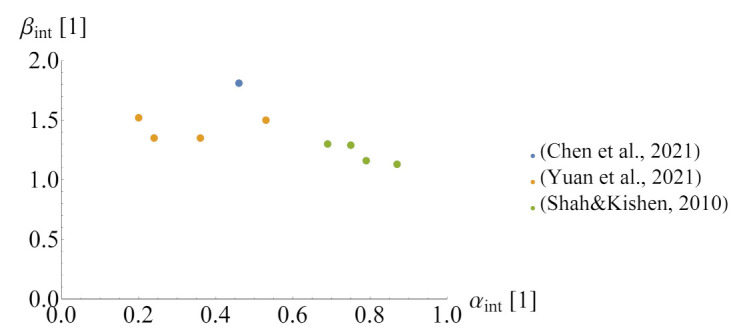
The relationship between the bond efficiency coefficient and a decrease in the fracture energy.

The fracture energy in mode I is similar to the energy in mixed mode I-II conditions [45], whereas in a pure mode II, it is much larger—about 20 times more [46]. The fracture energy, when the interface is under simultaneous compression and shear, is even greater, since, after the crack formation, the interface is capable of transmitting shear stresses due to the shear-friction mechanism [42,47,48]. Tests for a concrete-to-concrete interface, in which the fracture energy was determined in addition to its strength, are scarce, so the assumption of the correct value of this parameter is not an easy task. The following relation can be assumed between the fracture energy of the interface ($G_{f,int}$), the bond efficiency coefficient ($\alpha_{int}$), and the fracture energy of rgw weaker concrete ($G_{f,weak}$):

(16) $$G_{f,int}=\frac{\alpha_{int}}{\beta_{int}}\;G_{f,weak}$$

where: $\beta_{int}$—the coefficient that can be related to $\alpha_{int}$. These relations obtained by three research teams ([26,34,37]) are summarised in Figure 7. After analysing the data, it can be concluded that the value of coefficient β should be taken from 1.2 to 2.0. In case of a lack of experimental results, the fracture energy of weaker concrete in modes I and II can be estimated with the following formulas [36,46]:

(17) $$G_{f,I}=10{\left(d_{max}\right)}^{0.33}{\left(f_{cm}\right)}^{0.33},$$

(18) $$G_{f,II}=0.429{\left(d_{max}\right)}^{0.1416}{\left(f_{cm,cube}\right)}^{0.3042},$$

where: $d_{max}$—the maximum aggregate size (in (mm)), and $f_{cm,cube}$—the cubic compressive strength (in (MPa)).

The whole calibration strategy for a concrete-to-concrete interface was summarised in Table 7. The viscous regularisation was used to obtain a stable model response in the post-peak regime. It is worth mentioning that in the CAE module of the Abaqus code, this parameter has to be introduced via the “Assign Element Type” window, which is not in the “Property” module.

The **concrete** region was modelled with C3D8 continuum elements with selective integration [15]. The concrete damage plasticity (CDP) was chosen as a constitutive model for concrete. The version without the scalar damage parameter was assumed since only monotonic loads were analysed. The theoretical background of this model was presented in references [50,51]. The model has gained significant popularity and was discussed in detail in many references, e.g., [52,53,54,55], so its thorough description could be omitted in this study.

In general, the model is formulated in the framework of the theory of plasticity with an additive strain tensor decomposition:

(19) $$\sigma =D^{el}\left(\epsilon -\epsilon^{pl}\right),$$

where: ϵ and $\epsilon^{pl}$—total and plastic strain tensors, respectively, and $D^{el}$—the elasticity tensor for isotropic material. The CDP model uses a yield criterion proposed by Lubliner [50] and isotropic strain hardening/softening. The flow is governed by a non-associated flow rule. As the plastic potential, the smoothed Drucker–Prager cone is assumed. The hardening/softening in tension is controlled by equivalent plastic strain in tension (PEEQT according to the manual [15]), whereas, in compression, another equivalent plastic strain is introduced (PEEQ according to [15]). Distribution of these scalars can be used to analyse which regions of the model are cracked (PEEQT) and which regions are crushed under compression (PEEQ) during the loading history of the analysed specimen. The parameters that have to be entered into the programme are summarized in Table 8.

In all presented case studies, the shape of the loading device was modelled precisely. In order to reduce the computational effort, they were discretized with R3D4 rigid elements. Between the surfaces of the loading device and the specimens, the contact with Coulomb friction was assumed with the friction coefficient equal to 0.6. Due to convergence issues, the simulations were displacement-driven, i.e., as the boundary conditions, displacements of loading devices were assumed.

The non-linear problem was solved using an incremental-iterative Newton–Raphson algorithm. Two default convergence criteria were used, which are precisely described in reference [3]. The tolerance for unbalanced forces was assumed as equal to 2%, whereas the tolerance for displacement correction vector was set to 1% (default values).

## 4. The Validation of the Proposed Strategy

### 4.1. CS1—Tests with One Cohesive Element

**Figure 8:**
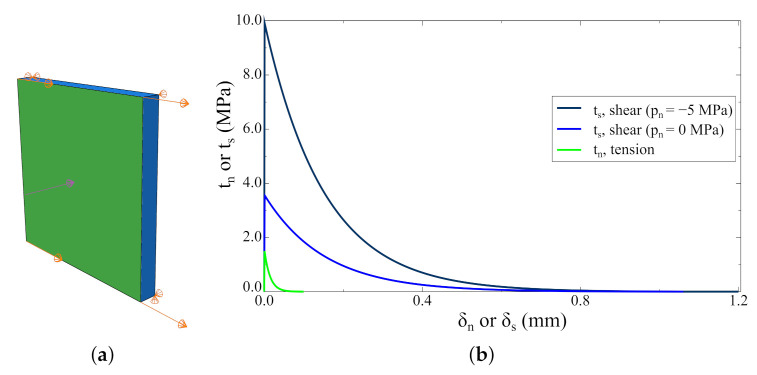
One element tests: (**a**) the numerical model; (**b**) the results for three different loading histories.

The first example concerns a numerical model consisting of one C3D8 continuum and one COH3D8 interface element—see Figure 8. Mechanical parameters adopted for constitutive models can be found in Table 9. Results for three loading paths are presented:

- displacement-driven tension (pure mode I), i.e., $\delta_{n}>0$, $\delta_{s}=\delta_{t}=0$,
- displacement-driven pure shear (pure mode II), i.e., $\delta_{n}=\delta_{t}=0$, $\delta_{s}>0$,
- at the first step, the pressure that induces compressive normal traction; at second-step displacement—driven shear, i.e.,  $\delta_{n}=\delta_{t}=0$, $\delta_{s}>0$.

The results of all performed analyses are shown in Figure 8b in one plot to highlight the differences in the dissipated energy in mode I and mode II. Moreover, it is clearly visible that the model predicts an increase in the shear strength due to a normal compressive traction. Some discrepancy between the model prediction and the real behaviour of a concrete interface is the softening branch shape in compressive states. Usually, it is assumed that the interface has residual strength in the case of a shear under compression due to shear forces [40,42,57], whereas the model predicts that shear traction stresses tend to be zero. On the other hand, the additional amount of energy is dissipated in this case, which may be related to shear forces. Summarising, the model behaviour is consistent with the assumptions from Section 3.

### 4.2. CS2—Simulation of Three-Point Bending of a Notched Beam

**Figure 9:**
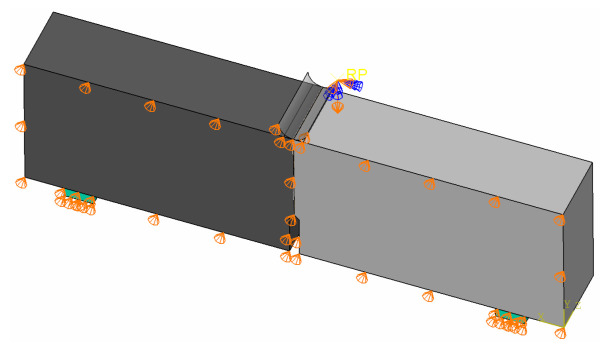
The geometry of the notched beam with assumed boundary conditions.

**Figure 10:**
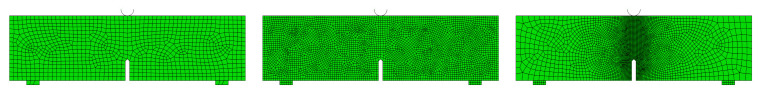
Meshes adopted in the analyses. Mean mesh size in the vicinity of the notch (**left** to **right**): 10 mm, 5 mm, and 2 mm.

The next example is a simulation of a three-point bending test of a beam with a notch described in Section 2.1. Due to the symmetry of the specimen, half of the beam was modelled. The qeometry of the model with assumed boundary conditions (symmetry condition on the symmetry plane and simple supports) is shown in Figure 9. Mesh dependency studies were performed assuming three mesh densities in the vicinity of the notch and the interface: 10, 5, and 2 mm—see Figure 10.

**Figure 11:**
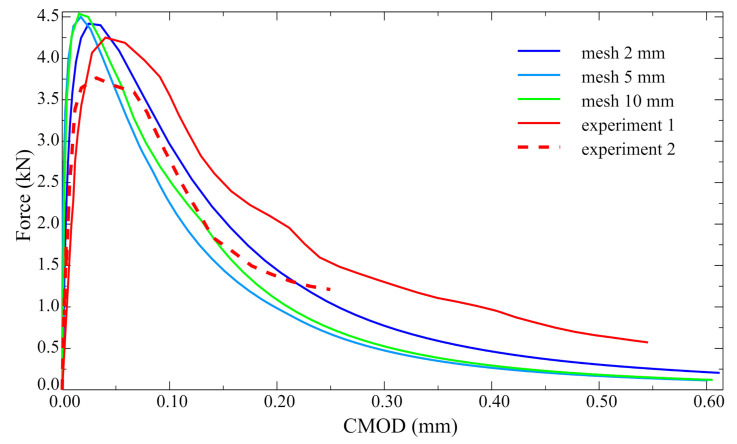
The plot of force vs. CMOD relation.

**Figure 12:**
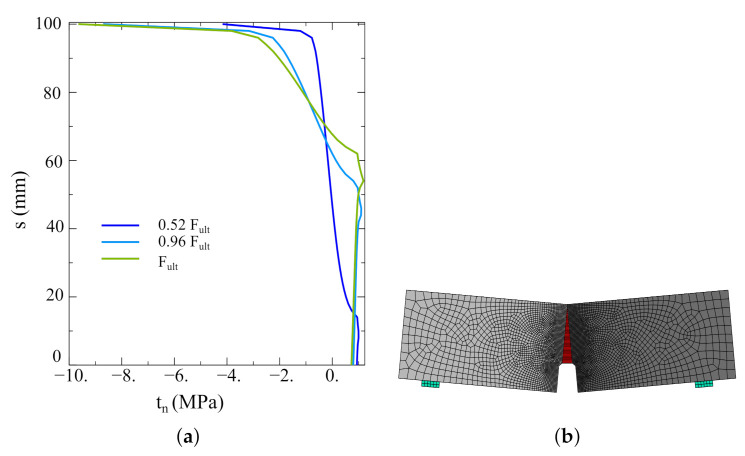
(**a**) Normal traction distribution along with the interface for different load levels. (**b**) Deformed model.

The results of the performed analyses are shown in Figure 11 as force–crack-mouth-opening-displacement (CMOD) curves. They were compared with the experimental outcomes reported in reference [26]. The best agreement was obtained for the densest mesh (2 mm). The little discrepancies may be caused by the non-homogenous distribution of concrete strength. In Figure 12, the distribution of normal traction in the interface is shown for three load levels ($0.52\;F_{ult}$, $0.96\;F_{ult}$, and $F_{ult}$) as well as a deformed mesh.

### 4.3. CS3—Simulation of Own Tests

**Figure 13:**
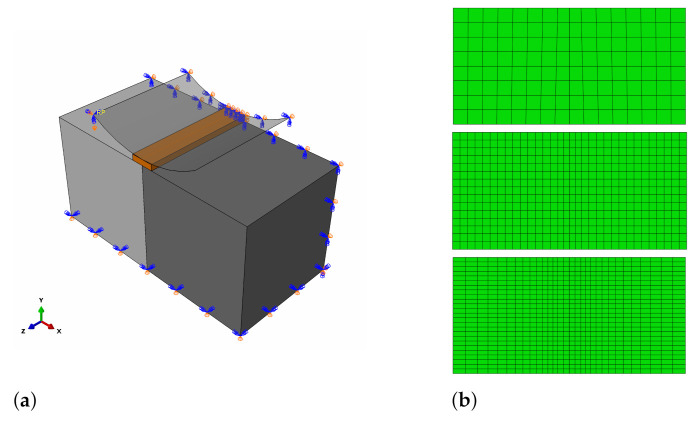
(**a**) Model of the splitting test. (**b**) Meshes adopted in the analyses (from the top): 10 mm, 5 mm, and 2 mm.

**Figure 14:**
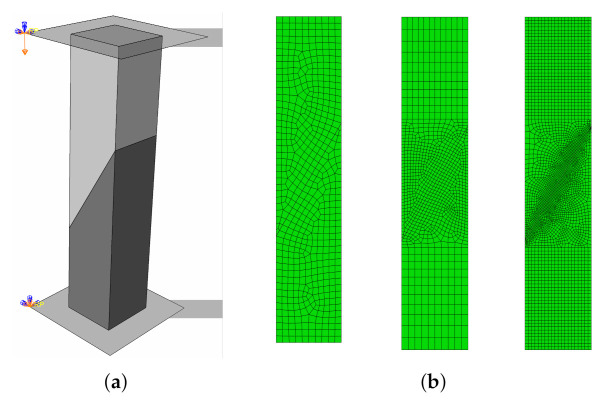
(**a**) Model of slant-shear test. (**b**) Meshes adopted in the analyses (respectively): 10 mm, 5 mm, and 2 mm.

The following case study concerns the simulation of our own laboratory tests: splitting and slant-shear of a concrete prism. A detailed analysis of the one-material splitting test, sometimes called “a Brazilian test,” can also be found in reference [58]. The models and meshes adopted in the analyses are shown in Figure 13 and Figure 14. In the case of the splitting test, the double symmetry of the task was used—the quarter of the specimen was considered. As in the previous CS, three mesh densities next to the interface were studied: 10, 5, and 2 mm. The test stand was modelled precisely, e.g., the exact geometry of a loading device with a pad was reproduced.

**Figure 15:**
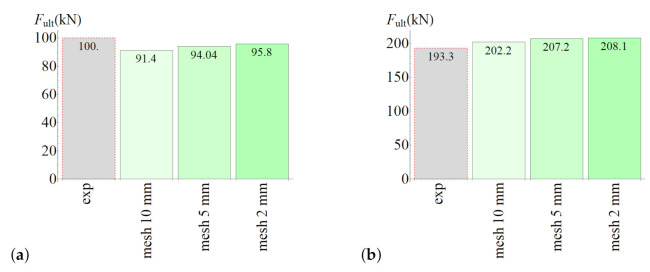
(**a**) Results of the splitting test simulation. (**b**) Results of the slant-shear test simulation.

The ultimate forces obtained from laboratory tests and numerical analyses are shown in Figure 15. The model for the splitting test slightly underestimated the ultimate force, whereas the model for the slant-shear test predicted value that was slightly too large. However, the differences were not too great (4–8%).

**Figure 16:**
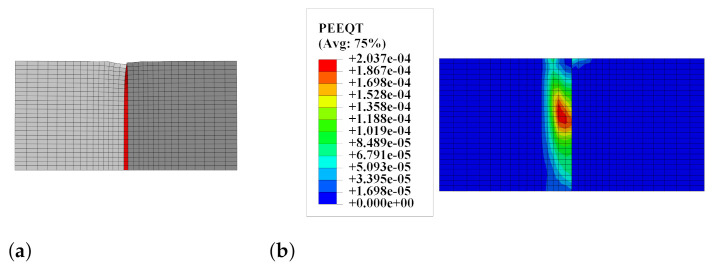
(**a**) The splitting test—deformed mesh. (**b**) The splitting test—a map of the equivalent plastic strain in tension PEEQT.

A deformed mesh and a map of equivalent plastic strain in tension (PEEQT according to the programme documentation [15]) for the splitting probe are shown in Figure 16. The plastic strains occurred in the weaker concrete, so the stresses during the analysis exceeded its tensile strength. This observation explains the increased possibility of mixed failure mode (adhesive–cohesive) in the case of the interface with a large value of $\alpha_{int}$ loaded in accordance with the standard procedure [39,59]. This is caused by problems with the uniform loading of both parts of the bi-material specimen.

**Figure 17:**
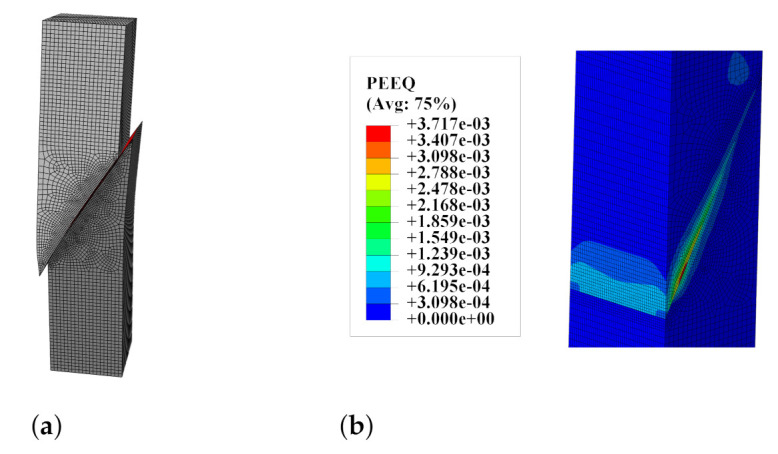
(**a**) The slant-shear test—deformed mesh. (**b**) The slant-shear test—map of the equivalent plastic strain in compression PEEQ.

A deformed mesh and map of equivalent plastic strain in compression (PEEQ according to the programme documentation [15]) for the slant-shear test are shown in Figure 17. The failure mode was reproduced correctly. Concentrations of plastic strains near the sharp edge explain the characteristic chips that appeared after the strength test of such specimens.

**Figure 18:**
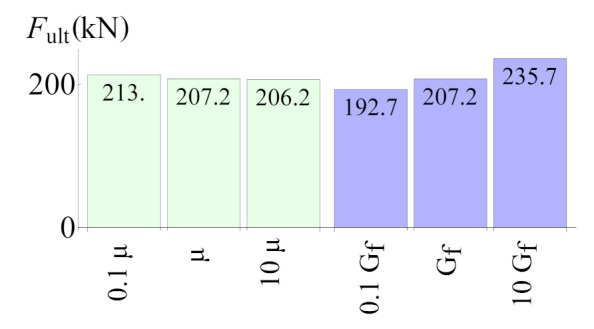
The slant-shear test—results of parametric studies.

Figure 18 summarises the result of the parametric studies performed using the slant-shear test model. They were performed in order to asses the influence of the viscous parameter μ, which has no clear physical meaning and which was introduced to stabilise the incremental-iterative process. The fracture energy of an interface ($G_{f,I}$ and $G_{f,II}$ were equally increased/decreased) was estimated according to Section 3. The parametric studies revealed that parameter μ has a very small impact on the results. Conversely, the ultimate force prediction strongly depends on the fracture energy, which proves that the issue of its proper estimation is of great importance.

### 4.4. CS4—Simulation of Santos and Julio Tests

**Figure 19:**
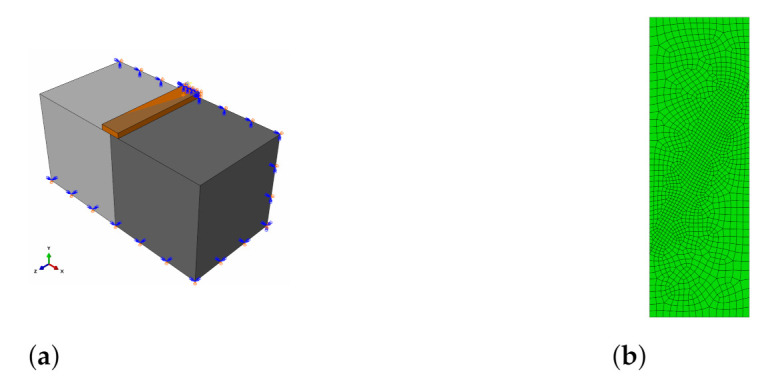
(**a**) Model of splitting test. (**b**) Mesh adopted in the analysis—element size in the vicinity of the interface—5 mm.

The last CS concerns the simulation of Santos and Julio [21,25] tests described in Section 2.3. Since our own tests followed the same testing programme, the boundary conditions applied in the analysis were almost the same except for the method of splitting the specimen loading. The specimen was loaded with a plain, narrow metal surface, not with a cylindrical-shaped device—see Figure 19a. The mesh of size 5 mm was used because of a small change in the ultimate force prediction in comparison to a 2 mm mesh in the previous CS. The mesh for a slant-shear test is shown in Figure 19b because of a different specimen size (CS3—100 mm × 100 mm × 500 mm; CS4—150 mm × 150 mm × 450 mm).

**Figure 20:**
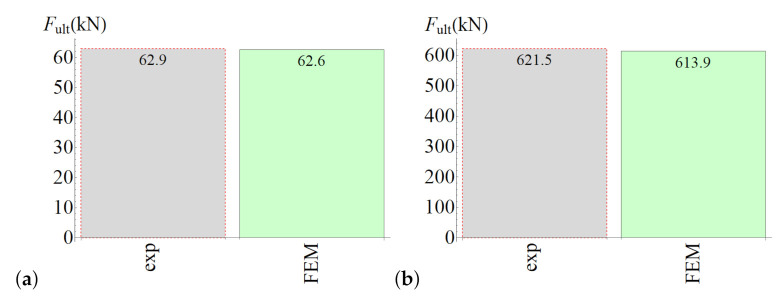
(**a**) Results of the splitting test simulation. (**b**) Results of the slant-shear test simulation.

A comparison of results obtained with the numerical analysis and from experimental tests is shown in Figure 20. The agreement between them was very good (with differences not greater than 2%), which proves the reliability of the formulated modelling strategy.

**Figure 21:**
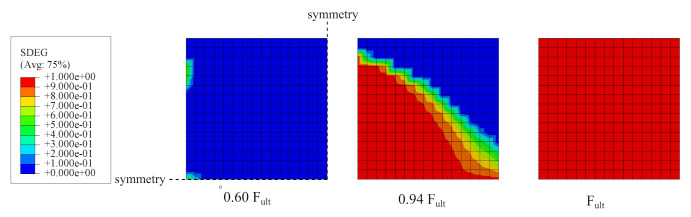
Results of the splitting test simulation—evolution of the degradation parameter in the interface.

In Figure 21, the evolution of the scalar degradation parameter *D* for an interface is presented as maps for three load levels: 0.6 $F_{ult}$ (initiation of damage), 0.94 $F_{ult}$, and $F_{ult}$.

**Figure 22:**
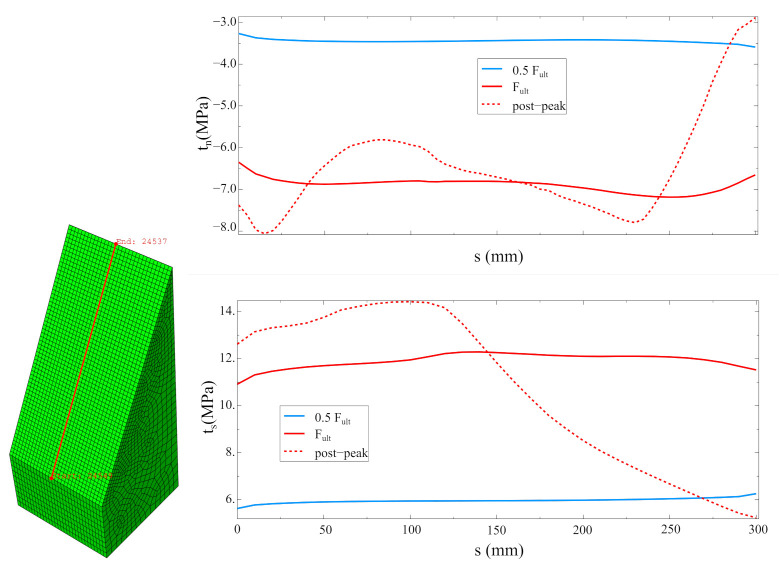
Distributions of normal and tangential tractions along the selected cross-section.

Traction distributions along the selected cross-section (in the middle of the specimen) are shown in Figure 22. In the elastic stage (0.5 $F_{ult}$), stress concentrations next to the specimen edges, caused by its inhomogeneity, can be observed. For the ultimate force ($F_{ult}$), the delamination of the specimen is progressing (decrease in tractions at the left edge). In the post-peak regime, the rest of the concrete contact is additionally loaded (increase in tractions), so the proper definition of the strength envelope is crucial also in this stage. It is worth mentioning that in real structures the progressive delamination of two concrete interfaces rarely leads to reaching the load capacity of the whole structural system.

## 5. Discussion

The strategy for adjusting an interface model available by default in the Abaqus code to the behaviour of concrete-to-concrete interface was proposed in Section 3. It was based on cohesive elements with the damage-elastic traction–separation constitutive model, which should be used to model initially rigid and thin connections between layers made of different concretes [13,15,40]. The standard model was enhanced using the simple USDFLD user procedure, which enabled us to introduce the strength envelope, which is dependent on a normal traction value. The model took into account the different fracture energy values for modes of fractures I and II. The proposed approach was verified and validated with four case studies concerning: one element test, simulation of three-point bending of the bi-material notched beam [26], simulation of two series of splitting, and slant-shear tests—our own pilot research and the research made by Santos and Julio [21,25]. The results of the performed analyses are summarised in Table 10. The ratio of the ultimate force predicted by the FEM model and obtained as an outcome of a laboratory test was calculated for CS2-CS4. Its mean value was 1.01, and the CoV was 5%, which proves the the accuracy of the proposed modelling strategy.

Besides, the fully non-linear simulation of the most popular laboratory tests aimed at determining the interface strength gave a better insight in the specimen’s behaviour under loading. The simulation for the three-point bending test of the notched beam showed the progressive delamination in the interface, cf. Figure 12. Such a phenomenon can be examined using modern optical measurement techniques [27]. On the contrary, such techniques fail in monitoring fracture process for other analysed tests (the splitting and slant-shear tests) due to the brittle failure mode of such specimens [23]. Therefore, the numerical methods are appropriate tools to understand their behaviour in depth. In the present research, the characteristic features of their failure modes were reproduced, such as the mixed failure mode for cubic specimens in the splitting test—see Figure 16—as well as the formation of chipping regions in the slant-shear test—see Figure 17. Moreover, the traction distributions along the interface in different stages of the tests were shown—see Figure 22.

The limitation of the presented approach is the inability of the model to cover the residual strength envelope caused by the shear–friction phenomenon and the irreversible (plastic) slip since the default traction–separation material model was formulated in the framework of damage-elasticity without the plastic part. These issues can be overcome by the implementation of the traction–separation law within the UMAT or UEL user procedure [57].

## 6. Conclusions

The article presented the complete strategy for calibration of the concrete-to-concrete interface model in the Abaqus code. The default traction–separation implemented in this system does not take into account the strength envelope for such interfaces. This issue was overcome with the USDFLD user subroutine coded in FORTRAN, which enabled us to relate a shear strength with tractions normal to the interface. Different values of fracture energy in fracture modes I and II were also included. The results presented in the article can be used in the analyses of complex composite structures made of concrete layers cast at different times as well as in the assessment of various methods of repairing deteriorated structures. The presented modelling approach can also be useful in the simulation of masonry structures—the interface model can be easily adjusted to the behaviour of mortar [41].

Furthermore, very detailed simulation of the most popular strength tests’ methods for concrete-to-concrete interfaces was presented in four case studies. The following observations can be made on their basis:

- The method of loading specimens, which is fully consistent with the standards for splitting tests [39], can increase the possibility of obtaining cohesive or mixed failure modes (see Figure 16), so the modification of the test procedure introduced by Santos and Julio [25] seems to be reasonable.
- In the case of small specimens, the mesh density of 5 mm was sufficient to obtain satisfactory accuracy of the FEA results.
- The numerical model was able to cover the chipping of sharp edges for slant-shear specimens (see Figure 17).
- The value of fracture energy has a noticeable influence on the ultimate force predicted by the model (see Figure 18), so this issue should be experimentally studied in more depth due to the small amount of the experimental data.
- The traction stress distribution is not homogenous along the interface during the whole loading history in the case of slant-shear specimens made of concrete, which are of different classes (see Figure 22 and reference [21]). Consequently, the interface strength characteristics determined according to Equations (3) should be corrected in order to take into account stress concentrations present in the prism specimens.

Further studies will be concentrated on the determination of the fracture energy for different concrete-to-concrete interfaces (different concrete classes and surface preparation) and its dependency on the bond efficiency coefficient. Additionally, an attempt will be made to implement more of the sophisticated interface models—examples of such models can be found in references [40,42,57]. 

## Figures and Tables

**Table 1:** The main laboratory tests outcomes from reference  [26]. n/a—not applicable, n/t—not tested, n/g—not given.

Tested Item	$f_{cm,cube}$	CoV	$f_{ctm,split}$	CoV	$f_{ctm,flex}$	CoV	$G_{f}$	CoV
	(MPa)	(%)	(MPa)	(%)	(MPa)	(%)	(N/m)	(%)
Low-strength concrete (LSC)	68.3	n/g	3.7	n/g	3.99	6.3	225.8	11.3
High-strength concrete (HSC)	34.4	n/g	2.7	n/g	6.40	6.2	271.2	5.4
LSC-to-HSC interface	n/a	n/a	n/t	n/t	1.84	7.6	57.4	25.6

**Table 2:** Mix compositions.

Mix Composition	Concrete A	Mix Composition	Concrete B
Compound	(kg/m${}^{3}$)	Compound	(kg/m${}^{3}$)
Cement I 42.5 R	350	Cement I 42.5 R	506
Vistula sand 0/2 mm	687	Fly ash	83
Gravel 2/8 mm	465	Vistula sand	620
Gravel 8/16 mm	706	Amphibole 2/8 mm	889
Water	163	Amphibole 2/8 mm	279
Superplasticizer	3.25	Water	159
		Superplasticizer	9.3

**Table 3:** Results of the strength tests for the concretes.

Tested Concrete	$f_{cm,cube}$	STD	CoV	$f_{ctm,split}$	STD	CoV
	(MPa)	(MPa)	(%)	(MPa)	(MPa)	(%)
Concrete A	46.6	2.8	6.0	3.20	0.07	2.3
Concrete B	79.4	1.6	2.0	8.10	0.12	1.5

**Table 4:** Results of the strength tests for the interface.

Test	Results					
**Test**	$f_{\mathrm{ctm},\mathrm{split}}$ **(MPa)**	**STD (MPa)**	**CoV (%)**	**Failure Mode**	$\alpha_{\mathrm{int}}$	
Splitting	2.8	0.3	10.0	Mixed	0.88	
	$F_{\mathrm{ult}}$ **(kN)**	**STD (kN)**	**CoV (%)**	$\sigma_{\mathrm{ult}}$ **(MPa)**	$\tau_{\mathrm{ult}}$ **(MPa)**	**Failure Mode**
Slant-shear	193.3	33.1	17.1	4.83	8.37	Adhesive

**Table 5:** The main laboratory tests’ outcomes for the used concretes in reference [25].

Tested item	$f_{cm,cube}$ (MPa)	STD (MPa)	CoV (%)
Substrate	79.3	4.8	6.0
Added	66.4	2.1	3.2

**Table 6:** The main laboratory tests’ outcomes for the interface in reference [25]. n/a—not applicable, n/t—not tested.

Test	Results					
	$f_{\mathrm{ctm},\mathrm{split}}$ **(MPa)**	**STD (MPa)**	**CoV (%)**	**Failure Mode**	$\alpha_{\mathrm{int}}$	
Splitting	1.8	0.3	15.4	Adhesive	0.46	
	$F_{\mathrm{ult}}$ **(kN)**	**STD (kN)**	**CoV (%)**	$\sigma_{\mathrm{ult}}$ **(MPa)**	$\tau_{\mathrm{ult}}$ **(MPa)**	**Failure Mode**
Slant-shear	621.5	97.7	15.7	6.9	12.0	Adhesive

**Table 7:** The calibration of a traction–separation model for a concrete-to-concrete interface—main assumptions.

Symbol	Parameter	Assumed Value/Formula	Reference
	**Elasticity**		
$K_{nn}$	Stiffness in normal direction	$E_{weak}$	[13]
$K_{ss}$, $K_{tt}$	Stiffness in tangential directions	$K_{ss}=K_{tt}=G_{weak}$	[13]
*t*	Interface thickness	t=0.005a	[13]
	**Damage Initiation Criterion**		
$f_{t}$	Tensile strength (mode I)	$f_{t}\approx 0.9\;f_{ctm,split}$	[49]
$f_{sh}$	Shear strength (mode II)	$f_{sh}\left(t_{n}\right)$ acc. to Carol’s Formula (9), USDFLD	[42]
	**Damage Evolution Rule and Viscous Regularisation**		
	Softening type	Exponential shape	
α	Parameter of exponential function	≈7	[44]
$\delta_{m}^{fail,I}$	Failure separation in mode I	Sequentially acc. to: (16), (17), (15)	[36]
$\delta_{m}^{fail,II}$	Failure separation in mode II	Sequentially acc. to: (16), (18), (15)	[46]
μ	Viscosity parameter	0.0001	

**Table 8:** The calibration of the CDP model—main assumptions. (*)—strength substituted in MPa and dimensions in mm.

Symbol	Parameter	Assumed Value/Formula	Reference
	**Elasticity**		
*E*	Young’s Modulus	$E_{cm}=22,000{\left(f_{cm}\right)}^{0.3}$ (*)	EN 1992-1-1 [56]
ν	Poisson’s ratio	0.2	EN 1992-1-1 [56]
	**Ultimate surface**		
$f_{cm}$	Uniaxial compressive strength	$f_{cm}\approx 0.8\;f_{cm,cube}$	EN 1992-1-1 [56]
$f_{ctm}$	Uniaxial tensile strength	$f_{ctm}\approx 0.9\;f_{ctm,split}$	[49]
$\frac{f_{b0}}{f_{c0}}$	Ratio of biaxial to uniaxial compressive strength	1.16	[15]
$K_{c}$	Rarameter controlling the shape of deviatoric section	0.667	[15]
	**Hardening/Softening Rule**		
$f_{cy}\left(\epsilon^{pl}\right)$	Hardening rule in compression	EC2 parabola	EN 1992-1-1 [56]
$G_{f}$	Fracture energy in tension	$G_{f}=10{\left(d_{max}\right)}^{0.33}{\left(f_{cm}\right)}^{0.33}$ (*)	JSCE [36]
	**Plastic Potential and Viscoplastic Regularisation**		
ψ	Dilatancy angle	$30^{\circ }$	[52]
$\epsilon_{0}$	Eccentricity	0.1	[15]
μ	Viscosity parameter	0.0001	

**Table 9:** Parameters assumed in the case studies.

	CS1 & CS4	CS2	CS3
**CDP—weaker concrete**			
*E* (GPa)	36.3	29.8	32.6
ν (1)	0.2	0.15	0.2
0 $f_{cm}$ (MPa)	53.1	27.5	37.3
$f_{ctm}$ (MPa)	3.9	2.43	2.86
$G_{f}$ (N/m)	98	225	140
**CDP—stronger concrete**			
*E* (GPa)	38.3	36.6	38.3
ν (1)	0.2	0.15	0.2
$f_{cm}$ (MPa)	63.5	54.6	63.5
$f_{ctm}$ (MPa)	4.23	3.3	7.3
$G_{f}$ (N/m)	104	270	154
**Interface**			
$K_{nn}$ (GPa)	36.3	29.8	32.6
$K_{tt}$ or $K_{ss}$ (GPa)	15.1	13	13.6
*t* (m)	0.001	0.001	0.001
$f_{ctm}$ (MPa)	1.6	0.95	2.55
*c* (MPa)	4.5	1.9	6.6
fi (deg)	50	40	40
$G_{f,I}$ (N/m)	22.4	57	37
$\delta_{m}^{fail,I}$ (m)	$9.86\cdot 10^{-5}$	0.000423	0.000102
$G_{f,II}$ (N/m)	539	1425	925
$\delta_{m}^{fail,II}$ (m)	0.001035	0.006477	0.001283

**Table 10:** Summary of the performed validation tests.

Case Study No.	$F_{ult,exp}$	$F_{ult,FEM}$	$\frac{F_{ult,FEM}}{F_{ult,exp}}$
CS2	4.19	4.418	1.05
CS3—splitting	100	95.8	0.96
CS3—slant-shear	193.3	208.1	1.08
CS4—splitting	62.9	62.6	1.00
CS4—slant-shear	621.5	613.9	0.99
		mean	1.01
		STD	0.05
		CoV	0.05

## Data Availability

The data presented in this study are available on request from the authors.
